# Adult Lead Poisoning Caused by Contaminated Opium: A Two-Year Longitudinal Follow-Up Study

**DOI:** 10.5334/aogh.3420

**Published:** 2021-09-09

**Authors:** Malihe-Sadat Hosseini, Amir Salimi, Scott Phillips, Nasim Zamani, Hossein Hassanian-Moghaddam

**Affiliations:** 1 Pharmaceutical Sciences Branch, Islamic Azad University (IAUPS), Tehran, Iran; 2 School of Medicine, Shahid Beheshti University of Medical Sciences, Tehran, Iran; 3 University of Colorado Anschutz Medical Campus, Rocky Mountain Poison & Drug Safety, Denver, CO and Washington Poison Center, Seattle WA, USA; 4 Social Determinants of Health Research Center, Shahid Beheshti University of Medical Sciences, Tehran, Iran; 5 Department of Clinical Toxicology, Loghman Hakim Hospital, Shahid Beheshti University of Medical Sciences, Tehran, Iran

## Abstract

**Background:**

A major episode of lead poisoning caused by lead-adulterated opium occurred in Iran in 2016. Patients were removed from exposure and treated with chelating agents. A subset of those patients was evaluated in this follow-up study to evaluate treatment efficacy in relation to patient outcome.

**Methods:**

Between March 2016 and December 2017, thirty-five male cases of lead poisoning due to ingestion of lead-adulterated opium were followed for two years. There are three patient groups: 1) those who abstained from opium use; 2) those who continued to use potentially contaminated opium; and 3) those who abstained from opium and were placed on maintenance therapy. Maintenance therapy included: methadone and opium tincture, offered by the Opioid Maintenance Therapy (OMT) clinics. Amongst the three patient groups Blood Lead Levels (BLL), complete blood count, and kidney and liver function tests were compared.

**Findings:**

The results of BLL, hemoglobin, hematocrit, and aspartate aminotransferase were significantly different between the admission time and follow-up. Of the three patient groups, no difference was detected in these measures.

**Conclusions:**

Treatment of lead poisoning combined with OMT proved an effective method to prevent recurrent lead poisoning.

## Background

Industrially, lead is a useful metal due to its low melting point, resistance to corrosion, and high malleability and ductility [1,2,3]. Workplace lead exposure may result in a rise of lead dust that is a risk for lead poisoning [1]. Humans can be exposed to lead through oral, inhalation, and to a lesser extent, dermal contact [4,5,6]. For an individual who has normal renal function, the kidneys excrete about 75% of all lead while the remainder is eliminated through the gastrointestinal tract. However, lead toxicity may occur when the lead body burden exceeds the individual’s ability to eliminate the metal [3,7].

Acute lead toxicity is infrequent, typically occurring in pediatric and occupational settings [1], while chronic toxicity is much more common and occurs from exposure to environmental pollutants [8]. Patients are often asymptomatic, but may present with nonspecific signs and symptoms including abdominal pain, constipation, anemia, irritability, short term memory impairment, myalgia, and neuropathies [9]. Delayed appreciation and treatment may lead to irreversible complications such as renal failure, encephalopathy, paralysis, and even death [10].

Lead toxicity is a significant public health hazard stemming from environmental pollution, especially in developing countries [5]. Recent Iranian studies reported lead toxicity among opium abusers [11,12,13]. Substance abuse (mostly opium and its derivatives) is recognized as one of the most serious public health and social threats in Iran due to its long, somewhat porous, borders with Afghanistan, the biggest producer of opium in the world [10,14]. In 2015, Iranian Drug Control Headquarters estimated 2.8 million people aged 15–64 were substance abusers [15]. Studies showed drug associated lead toxicity cases were determined in adulterated opium, marijuana, and methamphetamine [16,17]. Lead contamination was also found in approved medications [18]. In 2016, lead-adulterated opium caused an epidemic in Iran. Lead was added to drugs of abuse, including opium, in order to increase the product weight and thereby increasing profit [7,10,19,20,21,22].

Treatment of lead toxicity should focus on discontinuation of exposure, high calcium and iron diets, and (possibly) chelating agents including dimercaprol or British Anti-Lewisite (BAL), calcium disodium ethylenediaminetetraacetic acid (CaNa_2_ EDTA), dimercaptosuccinic acid (DMSA), and d-penicillamine, if indicated [21,23,24,25]. Although as many as 40,000 lead-poisoned patients were treated in Iran during that endemic [10], longitudinal follow-up studies have previously been unavailable.

During the endemic, this center treated approximately 1,000 inpatients. Herein, this follow-up subset from the 2016 epidemic undertook clinical and laboratory assessments to determine 1) if the prior lead exposure/poisoning treatment resulted in improvement of lab tests, and 2) if a difference existed among those who had discontinued opium ingestion or returned to opium use—including patients who participated in maintenance therapy after lead poisoning.

## Methods

### Study Design and Setting

35 patients who had lead toxicity secondary to adulterated opium consumption and were admitted to the Loghman Hakim Hospital between March 2016 and December 2017 were studied. On presentation, all cases had an initial blood lead level (BLL) and had received standard chelation based on availability, BLLs, and clinical manifestations including a) D-penicillamine, b) BAL and EDTA, c) EDTA and D-penicillamine, d) EDTA [11]. These patients were recruited for a prospective cohort study to determine if changing the route of opium consumption or quitting opium consumption affected BLL after 22 to 26 months.

### Participants

All opium-dependent, lead-poisoned patients admitted to the toxicology service between March 2016 and December 2017 with on-arrival measured blood lead concentrations were asked to participate in the study. Of the 83 eligible opium-dependent patients discharged, 35 agreed to enroll and participate in the study. There are three patient groups: 1) those who had quit and no longer used opium; 2) those who continued to use opium (that was possibly lead-contaminated); and 3) those who no longer used opium and instead used safe maintenance therapy, including methadone and opium tincture, offered by the OMT (Opioid maintenance therapy) clinics. Of the 83 eligible opium-dependent patients discharged, one was female and the remainder male. The majority of these prior cases were lost to follow-up. All 35 participants in this longitudinal cohort were male. Thus, no multi-gender analysis was possible.

Lead poisoning is defined as ‘possible’ if signs and symptoms were accompanied by a BLL value greater than 30 µg/dL at the time of diagnosis and treatment initiation [9]. Patients considered with opioid use disorder are those meeting the DSM-IV TR (Diagnostic and Statistical Manual of Mental Disorders 4th Edition Text Revision) criteria for substance dependence [20]. Exclusionary criteria include those patients with a history of occupational lead toxicity (e.g., employees of battery manufacturer or automobile radiator repair), non-opium lead exposure, or underlying systemic diseases (including renal insufficiency).

### Variables & data sources

Demographic data and baseline lab tests were extracted from inpatient files. Patients were queried on route of opium consumption during follow-up telephone calls. Follow-up lab test specimens were taken in final follow-up visits after one year. Blood lead levels were analyzed by atomic absorption spectrometry (AAS) method for evaluating BLL. Mean values replaced missing data (three to five cases in each group).

### Statistical analysis

To analyze the data, Statistical Package for Social Sciences (SPSS) software version 26 (IBM Incorporations, Chicago, Ill, USA) was used. Findings are reported as median and interquartile range (IQR) and frequency or percentage. Baseline, final, and change from baseline values for BLL, hemoglobin (Hb), hematocrit (Hct), blood urea nitrogen (BUN), creatinine (Cr), and aspartate aminotransferase (AST) values are compared amongst three groups by Independent-Samples Kruskal-Wallis test. Test value changes from baseline within groups are compared by Related-Samples Wilcoxon Signed Rank test. To better recognize the effect of the route of opium consumption on BLL and Hb values, Group 1 (those who had quit and no longer used opium), which had extremely small numbers that interfered with statistical analyses reliability and significance, was excluded. Regarding the two remaining groups, changes in BLL, Hb, Cr, BUN, Hct, and AST values by Mann-Whitney Test were compared. In-hospital chelating agent treatments were compared to see if initial BLL changed after follow-up applying independent-Samples Kruskal-Wallis test. A P-value of less than 0.05 was considered statistically significant.

### Ethics approval and consent to participate

This study has been performed in accordance with the Declaration of Helsinki. The local ethics committee at Shahid Beheshti University of Medical Sciences approved the study (Code 29646). Informed written consent was taken from all participants.

## Results

Of the 83 opium-dependent, lead-poisoned patients evaluated for study eligibility, none met exclusion criteria. Included in the analysis are 35 (42%) patients who consented to, participated in, and completed the study. The mean age is 50 ± 12 years (range: 30 to 79 years). The most common signs and symptoms were abdominal pain (94%), constipation (24%), weakness (16%), paresthesia (15%), nausea and vomiting (13%), and bone pain (10%). All cases are male. Group 1, 2, and 3 accounted for 3, 14, and 18 cases, respectively.

At baseline, mean participants’ BLL was 122 ± 47 µg/dL (range: 37 to 248 µg/dL). Mean hemoglobin, BUN, Cr, hematocrit, and AST were 9.8 ± 19 g/dL (range: 6.6 to 13.4 g/dL), 29.7 ± 17 mg/dL (range: 8 to 80 mg/dL), 1.1 ± 0.3 mg/dL (range: 0.7 to 2.4 mg/dL), 30.1 ± 5.8% (range: 21.3 to 39.5%), and 58.6 ± 33.9 U/L (range: 19 to 145), respectively. Age distribution is similar amongst groups (p = 0.3). There were no significant differences between the groups at baseline, or on follow-up regarding BLL, Hb, Cr, BUN, Hct, and AST values (***Table 1***).

**Table 1:** Comparison of lab values in different groups.


TEST LEVEL	GROUP	BASELINE MEDIAN (IQR)	FOLLOW-UP MEDIAN (IQR)	MEAN DIFFERENCE	WILCOXON p-VALUE

BLL (µg/dL)	1	128 (-^a^)	17.6 (-^a^)	–90.83	0.109

	2	110 (83–127.9)	39.01 (28.22–46.45)	–60.83	0.002

	3	128 (105.35–173.5)	29.8 (26.57–36.92)	–104.83	0.000

	KW* p-value	0.189	0.133	0.129	–

Hb (g/dL)	1	8.5 (-^a^)	16.9 (-^a^)	7.5	0.109

	2	10.27 (9.22–11.42)	15.65 (15.25–16.15)	4.76	0.001

	3	9.67 (8.05–11.12)	14.9 (13.7–15.77)	5.22	0.000

	KW p-value	0.276	0.144	0.113	–

Cr (mg/dL)	1	1 (-^a^)	1.13 (-^a^)	0.01	1

	2	1.06 (0.87–1.2)	1 (0.87–1.01)	–0.19	0.028

	3	1 (1.07–0.9)	1.05 (0.9–1.2)	0.03	0.254

	KW p-value	0.508	0.341	0.299	–

BUN (mg/dL)	1	30 (-^a^)	43 (-^a^)	–6.97	1

	2	29.73 (22.25–31.25)	28.5 (23.47–36.5)	1.49	0.683

	3	21.5 (15.75–33)	35.5 (24–40.25)	8	0.071

	KW p-value	0.342	0.515	0.780	–

Hct (%)	1	26.6 (-^a^)	45.5 (-^a^)	19.23	0.109

	2	32.6 (25.7–37.4)	44.9 (41.92–47.45)	11.77	0.003

	3	28.4 (24.6–35.05)	44.1 (41.5–45.77)	13.79	0.000

	KW p-value	0.283	0.6	0.159	–

AST (U/L)	1	112 (-^a^)	18 (-^a^)	–69.66	0.109

	2	53 (35–58.7)	21.8 (18–27.5)	–24.86	0.004

	3	46.5 (32–83.25)	25.5 (17.7–37.25)	–33.72	0.001

	KW p-value	0.473	0.705	0.461	–


^a^ Interquartile range (IQR) is not calculated for Group 1. KW = Kruskal Wallis.

**Figure 1:**
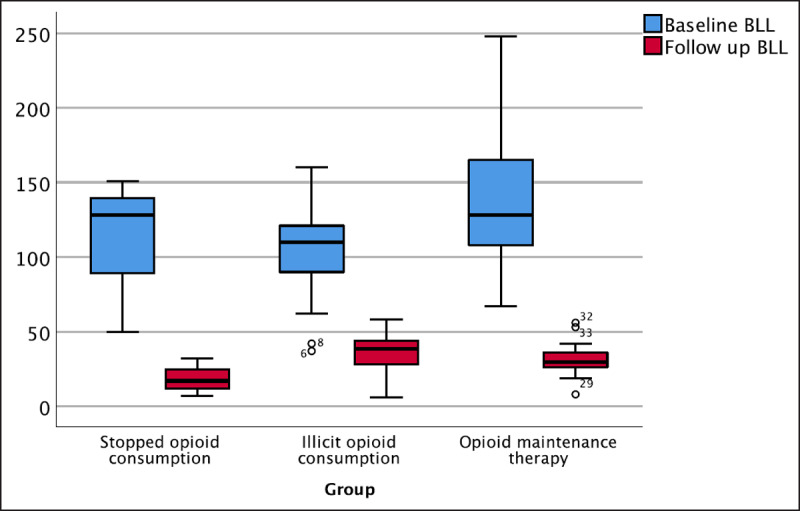
Baseline and follow-up BLL values in three distinct groups.

Follow-up BLL levels are significantly lower compared to those of the baseline values in all participants (p < 0.001), in Group 3 (p < 0.001), and in Group 2 (p = 0.002), but not among Group 1 (p = 0.109) (***Figure 1***). Only two participants, both of whom consumed ‘oral’ illicit opium, had BLL values increased from baseline.

**Figure 2:**
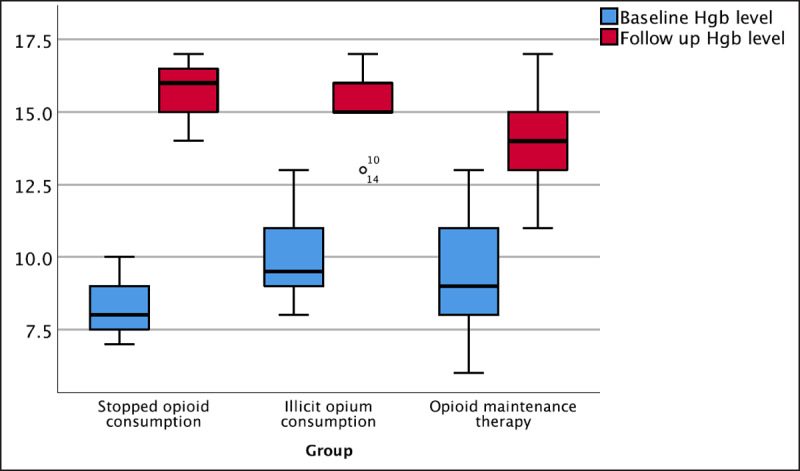
Baseline and follow-up hemoglobin values in three distinct groups.

Significant changes from baseline were seen in values for: Hemoglobin (increase), AST (decrease), Hct (increase); changes in Cr and BUN values were not significant. None of the participants showed a decrease in Hb values (***Figure 2***). Creatinine had increased in 12, decreased in 16, and not changed in 7 participants.

Mann-Whitney U test between Groups 2 and 3 (Group 1 was bypassed due to small sample size) showed a marginally significant difference in BLL value changes from baseline (p = 0.053). No significant difference was shown between Groups 2 and 3 regarding Hb (p = 0.547), Cr (p = 0.111), BUN (p = 0.505), Hct (p = 0.335), and AST values (p = 0.740).

***Table 2*** shows on-arrival BLL and administered chelating agents during hospitalization to see the possible superiority of each regimen. There were no significant differences among chelating agents in terms of BLL reduction.

**Table 2:** Blood lead level in administered chelating agents during hospitalization and follow-up.


TREATMENT	EDTA (n = 3)	D-PENICILLAMINE (n = 19)	BAL+EDTA (n = 9)	EDTA+D-PENICILLAMINE (n = 4)	KW TEST

*On-arrival BLL (µg/dL)	69.4 [50, –] (50, 125)	116.9 [86.7, 147] (37, 200)	108 [83.7, 175] (67, 200)	147.5 [101.2, 226] (90, 248)	0.277

*Follow-up BLL (µg/dL)	29.5 [6.1, –] (6.1, 39.4)	32.2 [26.9, 41.5] (7.1, 58.2)	29.1 [20.6, 33.9] (17.16, 53.4)	42.2 [31.8, 53.6] (29.1, 56.7)	0.272

*BLL difference (µg/dL)	63.3 [20.5, –] (6.1, 39.4)	78.5 [60.5, 115.1] (-15.9, 168.9)	77.5 [46, 105.8] (40.9, 176.8)	112.9 [60.7, 173.4] (45.7, 191.3)	0.480


* Median [IQR] (min, max); KW = Kruskal Wallis.

## Discussion

Between March 2016 and December 2017, a lead poisoning epidemic occurred in Iran due to lead-adulterated opium. This epidemic was identified both as a health hazard and a public health emergency and attracted decision-makers’ attention [21]. Historically, illicit opiates and opioids have been adulterated with various harmful substances including strychnine, paracetamol, and some heavy metals such as lead and thallium [5]. Halting exposure to the source of poisoning as well as patient education are the central components of lead toxicity management and reduction of the disease burden [23,26].

In order to eliminate lead exposure, patients were counseled to discontinue use of adulterated opium, and educated on the potential adverse effects of opium and lead poisoning. Additionally, patients were counseled about the benefits of a calcium-rich and iron-rich diet which would reduce lead absorption [26]. In an effort to reduce harm, OMT clinics provide opioid agonists in a therapeutic and controlled environment. There are over 7,000 OMT clinics in Iran to provide treatment to more than 500,000 (0.6% prevalence) opioid-dependent individuals [10]. In addition to reduce harm by providing lead-free opioids, OMT clinics were crucial in the control of the lead toxicity outbreak. In comparison, in the United States (population 328 million) there is an estimated 745,000 (0.2% prevenance) heroin users, and 10 million with opiate use disorder (3% prevenance), mostly related to prescription drug consumption [27].

Blood lead concentrations decreased significantly in all participants for Group 2 and Group 3, but not in Group 1 (p = 0.109). Decrease in BLL values in this group was noticeable, as well; however, interpretation was limited to three participants. The small number of participants in Group 1 (those who quit opium) caused unreliable p values in this group. In Group 1, a prominent decrease in BLL was observed; BLL median changed from 128 µg/dL at baseline to 17.6 µg/dL at final visit, with a mean decrease of more than 90 µg/dL. Management, including inpatient chelation therapy, education, and follow-up was efficient in this group of participants. No significant difference was seen between the methods each participant—who continued using substance (opium or maintenance therapy) or antidote—used during hospitalization. Thus, the lack of difference between the management approaches may be the effect of reducing exposure to lead. In Iran, raw opium is traditionally refined in boiling water through filtration and removing insoluble material [3], and so, it is possible that those who continued consuming illicit opium had refined it before consumption or switched dealers and thereby switched opium products [22].

Lead toxicity has a wide range of clinical manifestations including anemia, renal insufficiency, and neurologic damage that can be clinically assessed and followed up by lab tests [7,28,29]. CBC, BUN, Cr, and liver enzyme tests were performed in both the initial inpatient admissions and follow-up clinic visits. Hematologic indices showed that treatment protocols and follow-ups were effective [26]. Renal test results didn’t show significant changes. AST decreased significantly from abnormal baseline values to normal final follow-up visit values. During follow-up visits, and due to halting exposure to both chelating agents and lead (both of which are hepatotoxic), no liver enzyme rise was noted [22,30,31,32].

Although results revealed that the treatment protocols [26] were successful in treating and managing adulterated opium-induced lead poisoning, no significant difference was seen between groups and chelating agents administered during hospitalization. Thus, in discharged patients, the method of opium use had no effect on the outcome and exposure prevention could be responsible for the observed effects [9,33,34]. The lead epidemic did persuade some opium-dependent individuals to use safer opioids or to exercise caution when buying opium, to refine it before using, and to consume lesser amounts [3,22], all of which reduced exposure to lead-adulterated opium leading to insignificant differences among the study groups. Another potential reason for non-significant differences is our limited sample size; sample size affects p-value, making it is less likely to find a relationship [35].

There are other limitations that may have affected the study results: As a result of their low socioeconomic status, most opioid-dependent Iranians do not have regular follow-up visits. A robust three-group comparison was not possible due to the few number of patients who consented to participate in the study. Larger sample sizes for each of the three groups would be beneficial to better evaluate the comparisons and interpretations.

## Conclusion

The results suggest that our approach of reduced consumption, and that education, chelation, and dietary counselling are beneficial treatments for lead poisoning. Moreover, educating patients about the sources of lead in adulterated opium is helpful in reducing BLL and improving hematologic and hepatic injury irrespective of opium consumption method or even quitting consumption.

## Data Accessibility Statement

Available from corresponding author on reasonable demands.

## Abbreviations

AAS: Atomic absorption spectrometry; AST: aspartate aminotransferase; BAL: British Anti-Lewisite; BLL: blood lead levels; BUN: blood urea nitrogen; CaNa_2_ EDTA: Calcium disodium ethylenediaminetetraacetic acid; CBC: complete blood count; Cr: creatinine; DMSA: Dimercaptosuccinic acid; DSM-IV TR: Diagnostic and Statistical Manual of Mental Disorders 4th edition Text revision; Hb: hemoglobin; Hct: hematocrit; IQR: Interquartile range; OMT: Opioid maintenance therapy.

## References

[1] Flora G, Gupta D, Tiwari A. Toxicity of lead: A review with recent updates. Interdiscip Toxicol. 2012; 5(2): 47–58. DOI: 10.2478/v10102-012-0009-2 23118587 PMC3485653

[2] Azizi MH, Azizi F. Lead poisoning in the world and Iran. Int J Occup Environ Med. 2010; 1(2): 81–87. DOI: 10.5121/ijnsa.2010.2306 23022790

[3] Hayatbakhsh MM, Oghabian Z, Conlon E, et al. Lead poisoning among opium users in Iran: An emerging health hazard. Subst Abuse Treat Prev Policy. 2017; 12(1): 43. DOI: 10.1186/s13011-017-0127-0 28982369 PMC5629748

[4] Centers for Disease Control and Prevention. Adult blood lead epidemiology and surveillance–United States, 2003–2004. MMWR Morb Mortal Wkly Rep. 2006; 55(32): 876–879. 16915221

[5] Karrari P, Mehrpour O, Abdollahi M. A systematic review on status of lead pollution and toxicity in Iran; Guidance for preventive measures. Daru. 2012; 20(1): 2. DOI: 10.1186/1560-8115-20-2 23226111 PMC3514537

[6] Leroyer A, Hemon D, Nisse C, Bazerques J, Salomez JL, Haguenoer JM. Environmental exposure to lead in a population of adults living in northern France: Lead burden levels and their determinants. Sci Total Environ. 2001; 267(1–3): 87–99. DOI: 10.1016/S0048-9697(00)00762-2 11286218

[7] Salehi H, Sayadi AR, Tashakori M, et al. Comparison of serum lead level in oral opium addicts with healthy control group. Arch Iran Med. 2009; 12(6): 555–558. 19877747

[8] Saxena G, Flora SJS, Flora GJS. Environmental occurrence, health effects and management of lead poisoning. In: Casas J, Sordo J (eds.), Lead: Chemistry, Analytical Aspects, Environmental Impact and Health Effects. Amsterdam: Elsevier B. V. 2006; 158–228. DOI: 10.1016/B978-044452945-9/50004-X

[9] Shabani M, Hadeiy SK, Parhizgar P, et al. Lead poisoning; a neglected potential diagnosis in abdominal pain. BMC Gastroenterol. 2020; 20(1): 134. DOI: 10.1186/s12876-020-01284-1 32375657 PMC7201765

[10] Ghane T, Zamani N, Hassanian-Moghaddam H, Beyrami A, Noroozi A. Lead poisoning outbreak among opium users in the Islamic Republic of Iran, 2016–2017. Bull World Health Organ. 2018; 96(3): 165–172. DOI: 10.2471/BLT.17.196287 29531415 PMC5840624

[11] Zamani N, Hassanian-Moghaddam H, Bahrami-Motlagh H, Ahmadi S, Phillips S. Lead poisoning due to ingestion of lead-contaminated opium: A diagnostic study on patients’ imaging findings. J Trace Elem Med Biol. 2019; 55: 26–32. DOI: 10.1016/j.jtemb.2019.04.016 31345361

[12] Zamani N, Hassanian-Moghaddam H. Notes from the field: Lead contamination of opium – Iran, 2016. MMWR Morb Mortal Wkly Rep. 2018; 66(51–52): 1408–1409. DOI: 10.15585/mmwr.mm665152a4 29300718 PMC5758300

[13] Afshari R, Emadzadeh A. Short communication: Case report on adulterated opium-induced severe lead toxicity. Drug and Chemical Toxicology. 2010; 33(1): 48–49. DOI: 10.3109/01480540903127340 20001217

[14] Momtazi S, Noroozi A, Rawson R. An overview of Iran Drug Treatment and Harm Reduction Programs. In: el-Guelbaly N, Carrà G, Galanter M (eds.). Textbook of Addiction Treatment: International Perspectives. 2015; 543–554. Milano: Springer. DOI: 10.1007/978-88-470-5322-9_25

[15] Amin-Esmaeili M, Rahimi-Movaghar A, Sharifi V, et al. Epidemiology of illicit drug use disorders in Iran: Prevalence, correlates, comorbidity and service utilization results from the Iranian Mental Health Survey. Addiction. 2016; 111(10): 1836–1847. DOI: 10.1111/add.13453 27177849

[16] Busse F, Omidi L, Timper K, et al. Lead poisoning due to adulterated marijuana. N Engl J Med. 2008; 358(15): 1641–1642. DOI: 10.1056/NEJMc0707784 18403778

[17] Allcott JV, 3rd, Barnhart RA, Mooney LA. Acute lead poisoning in two users of illicit methamphetamine. Jama. 1987; 258(4): 510–511. DOI: 10.1001/jama.1987.03400040108032 3599348

[18] Lim D-Y, Kang W-Y, Ahn J-S, et al. Collective exposure to lead from an approved natural product-derived drug in Korea. Ann Occup Environ Med. 2019; 31: e20. DOI: 10.35371/aoem.2019.31.e20 31620297 PMC6779882

[19] Jalili M, Azizkhani R. Lead toxicity resulting from chronic ingestion of opium. West J Emerg Med. 2009; 10(4): 244–246. 20046241 PMC2791725

[20] Ghaemi K, Ghoreishi A, Rabiee N, et al. Blood lead levels in asymptomatic opium addict patients; a case control study. Emerg (Tehran). 2017; 5(1): e69. 28894784 PMC5585839

[21] Soltaninejad K, Shadnia S. Lead poisoning in opium abuser in Iran: A systematic review. Int J Prev Med. 2018; 9: 3. DOI: 10.4103/ijpvm.IJPVM_22_17 29416839 PMC5787876

[22] Alinejad S, Aaseth J, Abdollahi M, Hassanian-Moghaddam H, Mehrpour O. Clinical aspects of opium adulterated with lead in Iran: A review. Basic Clin Pharmacol Toxicol. 2018; 122(1): 56–64. DOI: 10.1111/bcpt.12855 28802093

[23] Masoodi M, Zali MR, Ehsani-Ardakani MJ, et al. Abdominal pain due to lead-contaminated opium: A new source of inorganic lead poisoning in Iran. Arch Iran Med. 2006; 9(1): 72–75. 16649384

[24] Gracia RC, Snodgrass WR. Lead toxicity and chelation therapy. Am J Health Syst Pharm. 2007; 64(1): 45–53. DOI: 10.2146/ajhp060175 17189579

[25] Golalipour MJ, Roshandel D, Roshandel G, Ghafari S, Kalavi M, Kalavi K. Effect of lead intoxication and D-penicillamine treatment on hematological indices in rats. International Journal of Morphology. 2007; 25(4): 717–722. DOI: 10.4067/S0717-95022007000400008

[26] Paeezi M, Zamani N, Hassanian-Moghaddam H, et al. Treatment of adult lead poisoning with D-penicillamine. Drug Metab Pers Ther. 2019; 34(2). DOI: 10.1515/dmpt-2019-0003 31188756

[27] U.S. Department of Health and Human Services. What is the U.S. Opioid Epidemic? https://www.hhs.gov/opioids/about-the-epidemic/index.html. Accessed on Aug 16, 2021.

[28] Golmohammadi T, Ansari M, Nikzamir AR, Safari Abhari R, Elahi S. The effect of maternal and fetal lead concentration on birth weight: Polluted versus non-polluted areas of Iran. Tehran University Med J (TUMJ). 2007; 65(8).

[29] Hegazy AA, Zaher MM, Abd El-Hafez MA, Morsy AA, Saleh RA. Relation between anemia and blood levels of lead, copper, zinc and iron among children. BMC Res Notes. 2010; 3: 133. DOI: 10.1186/1756-0500-3-133 20459857 PMC2887903

[30] Ahmadinejad M, Ahmadipour M, Divsalar K. Blood lead level in opiate addicts hospitalized in the intensive care unit of a trauma referral center in Kerman, Iran. Addict Health. 2019; 11(1): 11–17. 31308905 10.22122/ahj.v11i1.220 PMC6612236

[31] Mokhtarifar A, Mozaffari H, Afshari R, et al. Cholestasis and seizure due to lead toxicity: A case report. Hepatitis Monthly. 2013; 13(11). DOI: 10.5812/hepatmon.12427 PMC3860072 24348646

[32] Esmaeilzadeh A, Ganji A, Jafari M, Dehestani V. A case report of abdominal pain and increased transaminases due to lead poisoning. Medical Journal of Mashhad University of Medical Sciences. 2012; 55(2): 124–127.

[33] Kim H-C, Jang T-W, Chae H-J, et al. Evaluation and management of lead exposure. Ann Occup Environ Med. 2015; 27: 30. DOI: 10.1186/s40557-015-0085-9 26677413 PMC4681084

[34] Ettinger AS, Leonard ML, Mason J. CDC’s Lead Poisoning Prevention Program: A long-standing responsibility and commitment to protect children from lead exposure. J Public Health Manag Pract. 2019; 25(Suppl 1 Lead poisoning prevention): S5–S12. DOI: 10.1097/PHH.0000000000000868 PMC6320665 30507764

[35] Thiese MS, Ronna B, Ott U. P value interpretations and considerations. J Thorac Dis. 2016; 8(9): E928–E931. DOI: 10.21037/jtd.2016.08.16 27747028 PMC5059270

